# Postgraduate students' perception of current and future pre-clinical prosthodontics curriculum: A web-based survey

**DOI:** 10.6026/973206300211421

**Published:** 2025-06-30

**Authors:** Kritika Kar, Arti Shyam, Harsh Chansoria, Alka Gupta, Subhash Sonkesriya, Shivani Gupta

**Affiliations:** 1 Department of Prosthodontics and Crown & Bridge, Government College of Dentistry, Indore, Madhya Pradesh, India; 2 Department of Orthodontics and Dentofacial Orthopaedics, Sri Dharmasthala Manjunatheshwara College of Dental Sciences, Dharwad, Karnataka, India

**Keywords:** CAD/CAM, dental photography, implant prosthodontics, prosthodontics, 3D scanners

## Abstract

The perceptions of postgraduate Prosthodontic students regarding their preclinical curriculum in India are of interest. A web-based survey of 200 second - and third-year students highlighted a demand for a more contemporary and technologically integrated curriculum. Students found the current exercises to be either adequate or excessive, with casting procedures in fixed prosthodontics being the most challenging. The majority supported the inclusion of modern technologies, such as CAD/CAM systems and 3D scanners. The findings suggest that curriculum reform should focus on enhancing technological integration to better prepare students for clinical practice.

## Background:

Prosthodontics is crucial in dental education, focusing on restoring oral function, aesthetics, and patient well-being [1]. Preclinical training provides foundational knowledge and technical skills for clinical practice, emphasizing hands-on learning [2]. With advancements in digital technologies like CAD/CAM, 3D printing, and intraoral scanning, prosthodontic care is becoming more accurate and patient-friendly. To meet modern challenges, dental curricula must evolve, integrating these innovations and focusing on evidence-based, patient-centred care [3]. In light of these developments, it becomes imperative to revisit and realign existing preclinical prosthodontics curricula. The pace of change in dental technology demands that the training environment evolve to stay relevant to modern clinical practices. Moreover, incorporating emerging technologies and methodologies into the curriculum can significantly enhance the learning experience for students, preparing them to handle the complexities of modern prosthodontic cases [4]. Postgraduate students, transitioning to advance clinical training, offer valuable feedback on the effectiveness of the current curriculum. Their recent experiences with both undergraduate training and clinical practice provide crucial insights into strengths, weaknesses, and areas for improvement [5]. Their perspectives on integrating new technologies, such as CAD/CAM systems and virtual simulations, can inform curriculum developers on how to address gaps and incorporate emerging tools [6]. Additionally, as dental technology and patient cases become more complex, there is a growing need for more hands-on experience with tools such as digital impressions and implant prosthodontics during preclinical training to ensure readiness for clinical practice [7]. Therefore, it is of interest to explore postgraduate students' perceptions of the current curriculum and their expectations for future developments, with the aim of enhancing the relevance and effectiveness of prosthodontics education.

## Materials and Methods:

## Study design:

The investigators conducted across-sectional population survey among postgraduate students of prosthodontics in India. The questionnaire was administered using Google Forms; the link for the same was sent to most of the 2nd and 3rd year postgraduate students in India through varied media platforms like WhatsApp, Facebook Messenger, email and Instagram. The information was also collected and automatically transferred to a spreadsheet. Maximum of the questions were closed-ended, either dichotomous or in a multiple- choice format, which is actually specific and offers the participants a fixed range of answers and some interrogatives were open-ended, to acquire both quantitative and nominal data with freedom based on varied subjects covered in the post-doctoral prosthodontic course. The study required students to spend about 5- 7 minutes completing a questionnaire. The study was accepted using the approach outlined by Dillman.

## Participant selection:

The questionnaire had a total of 21 questions that were discussed with specialists in the field. The final and agreed questionnaire was sent to 200, 2nd and 3rd year postgraduate students in India between the time periods of along with invitation letter. Anonymity and confidentiality were assured. A total of responses were received within the specified period of time and were included for analysis. A completed questionnaire indicated the allowance to partake in the study and participants were able to decline any involvement at the beginning or withdraw at any time before the submission. There were no individual identifiers on the questionnaire. The participants were told, as mentioned in the invitation letter, that they're free to participate or not and that choosing not to participate won't be a disadvantage to them in any way. Responses in percentile to the provided questionnaire have been given in Table 1 (see PDF).

## Results and Discussion:

The pie chart shown in Figure 1 (see PDF) represents the distribution of postgraduate students based on their year of study. The chart provides insights from 200 responses, with 58.5% of respondents being in their second year (blue segment) and 41.5% in their third year (red segment). This data highlights a relatively larger proportion of second-year students compared to third-year students, potentially indicating that the survey reached a greater number of individuals earlier in their postgraduate program. The pie chart displayed in Figure 2 (see PDF) illustrates the year of post-graduation distribution of respondents in the survey. Out of 200 responses, 58.5% of respondents are in their 2nd year (represented by the blue segment) and 41.5% are in their 3rd year (represented by the red segment). This distribution indicates a higher representation of second-year postgraduate students, who are generally engaged with foundational courses and skills in prosthodontics, while third-year students are typically more involved in clinical practices and advanced techniques. The survey findings offer insights into the perceptions of students at varying stages of their postgraduate training. The pie chart displayed in Figure 3 depicts postgraduate students' perceptions regarding the number of preclinical exercises in the prosthodontics curriculum. Among the 200 respondents, 38.5% of participants indicated that the number of exercises is inadequate (represented by the orange segment), while 31.5% felt that the number of exercises is adequate (represented by the blue segment). On the other hand, 30% of students believed that the number of exercises is too extensive (represented by the red segment). This distribution suggests a notable variation in students' experiences, with a significant portion feeling that there are either too few or too many preclinical exercises. It highlights potential areas of concern for curriculum developers, indicating that finding an optimal balance in the number of exercises may improve student satisfaction and effectiveness in learning. The pie chart displayed in Figure 2 (see PDF) depicts postgraduate students' perceptions regarding the number of preclinical exercises in the prosthodontics curriculum. Among the 200 respondents, 38.5% of participants indicated that the number of exercises is inadequate (represented by the orange segment), while 31.5% felt that the number of exercises is adequate (represented by the blue segment). On the other hand, 30% of students believed that the number of exercises is too extensive (represented by the red segment). Figure 4 (see PDF) presents the distribution of postgraduate students' perceptions regarding the time required to complete preclinical exercises within their 3-year academic curriculum, based on 200 responses. A substantial portion, 43.5%, felt that 9 months (represented by the orange segment) was the time required to complete these exercises, while 24% (green segment) believed that it took 12 months. Additionally, 20.5% of students (red segment) felt 6 months was sufficient and 12% (blue segment) thought 3 months was enough. This data indicates that the majority of students view the duration for preclinical exercises as closer to 9 or 12 months, suggesting that students may feel that additional time is needed to adequately complete these essential tasks. These perceptions may guide curriculum developers in evaluating and possibly adjusting the duration allocated for preclinical training to better suit student needs and expectations.

Figure 5 (see PDF) shows the distribution of postgraduate students' perceptions regarding which preclinical prosthodontic exercise they find easiest and simplest to understand and work with, based on 200 responses. The largest proportion of respondents, 51.5% (represented by the red segment) and found removable partial dentures to be the easiest exercise. 36.5% (represented by the blue segment) considered complete dentures the simplest, while 8.5% (represented by the orange segment) found fixed partial dentures the easiest. Only a small percentage, 3% (represented by the green segment), found preclinical maxillofacial prostheses the most manageable exercise. This distribution suggests that students find removable partial dentures to be the most straightforward preclinical task, possibly due to their simpler conceptualization and fewer technical challenges compared to other exercises such as fixed partial dentures or complete dentures. The data indicates varying levels of comfort and ease with different prosthodontic procedures, which could reflect the students' familiarity or preferences in prosthodontic techniques at the preclinical level. Figure 6 (see PDF) presents a bar chart illustrating the types of articulators used by postgraduate students in their prosthodontic training, based on 200 responses. The Hanau Wide Vue (semi-adjustable) articulator was the most commonly used, with 60.5% (121 responses) of students indicating its use. The Mean Value articulator came in second, with 51% (102 responses) of students choosing this type. Whip Mix (semi-adjustable) was used by 21.5% (43 responses), while the Simple hinge articulator was chosen by 23% (46 responses). The Denar (semi-adjustable) articulator had the least representation, with only 12.5% (25 responses) of students using it. This data highlights the preference for semi-adjustable articulators, with the Hanau Wide Vue being the most widely used, reflecting its perceived efficiency and relevance in preclinical prosthodontics training. The varying choices of articulators could be influenced by the type of procedures being practiced or personal preferences of students and faculty. The results of the survey provide valuable insights into postgraduate students' understanding of occlusal schemes in complete dentures, which is a critical aspect of prosthodontic education. The majority of respondents, 74%, reported having acquired knowledge of different occlusal schemes, indicating that a substantial portion of students are exposed to and familiar with the key principles involved in occlusal relationships. However, a notable 17% of respondents did not possess this knowledge and 9% were uncertain, suggesting potential gaps in the curriculum or individual learning experiences that need further attention. Among the students who acknowledged their understanding of occlusal schemes, balanced occlusion was the most widely recognized, with 53.5% of respondents indicating familiarity with this scheme? Balanced occlusion is essential in complete denture fabrication, as it aims to distribute occlusal forces efficiently across the dental arches, promoting stability and function. Following balanced occlusion, monoplane occlusion was the second most commonly understood scheme, with 39.5% of students reporting familiarity. Monoplane occlusion is often considered a simpler and more stable approach, particularly in cases where there are significant concerns about the vertical dimension or patient comfort. Other occlusal schemes reported in the survey include lingualized occlusion (37%), neutrocentric occlusion (30.5%) and non-balanced occlusion (37.5%), with each scheme representing different philosophies regarding the relationship between the upper and lower teeth during function. Lingualized occlusion, which aims to emphasize the role of the mandibular incisors and the maxillary lingual cusps, is often employed in situations where there is a need for greater esthetic outcomes or better control over vertical dimension. Neutrocentric occlusion and non-balanced occlusion, on the other hand, reflect alternative approaches that are less commonly used but still relevant in particular clinical scenarios, such as in patients with specific prosthodontic needs. Interestingly, only 18.5% of students reported having a comprehensive understanding of all the occlusal schemes listed, which suggests that while individual schemes are relatively well understood, a holistic understanding of all approaches may be less common. This observation implies that there may be a need for greater emphasis on teaching and reinforcing the application of multiple occlusal schemes to allow students to make more informed decisions based on the specific requirements of their patients. These findings reflect the current state of postgraduate prosthodontic education, where foundational knowledge in occlusion is present but may vary in depth and breadth. The results underscore the importance of further enhancing curriculum content to ensure that students not only understand the commonly used occlusal schemes but also gain comprehensive knowledge of all available methods to better adapt to diverse clinical situations. By addressing these gaps, dental education can more effectively prepare students to address the complex challenges of complete denture fabrication in practice.

In terms of fixed partial denture preclinical exercises, the casting procedure emerged as the most difficult step, with 60% of participants expressing that they struggle with this particular part of the process. This could be due to the complexities involved in ensuring accurate and precise casting, which is critical for the successful creation of the denture. The next most challenging step was wax pattern fabrication, which 33.5% of the respondents found difficult. Wax patterning plays a crucial role in shaping the denture and its difficulty might stem from the need for fine detailing and the precision required achieving a well-fitted final product. Conversely, die cutting and ditching, which is an important step for separating the dental models from the molds, was seen as a relatively less challenging task, with only 15% of respondents reporting difficulty. The ceramic build-up, which involves layering ceramics to create the desired tooth shapes, was challenging for 25% of participants and finishing and polishing of the prostheses was found to be the easiest, with just 2.5% of respondents finding it difficult. These results highlight the varied skill sets required in prosthodontics, with casting and pattern fabrication being the most technically demanding aspects of the process. When it comes to the use of surveyors for removable partial denture design, the survey indicates that a majority of dental students (58%) rely on the surveyor for cast partial denture design, which involves examining the oral cavity to determine the ideal placement for dentures. This is a fundamental step in ensuring that the dentures fit well and function optimally. 17% of respondents use surveyors for acrylic partial dentures, which are typically more cost-effective but may require less precise planning compared to cast dentures. Furthermore, 20% of respondents use surveyors for both types of dentures, indicating a broad application of this tool across various types of partial denture designs. Only a small proportion (5%) does not use surveyors for any type of partial denture, which could reflect either a lack of access to this tool or preference for alternative methods. Regarding materials used in maxillofacial prostheses fabrication, Polymethyl Methacrylate (PMMA) resin was the most commonly chosen material, with 58.5% of respondents indicating its use. PMMA is a widely accepted material due to its strong mechanical properties and aesthetic qualities, making it ideal for prosthetics. RTV silicone followed as the second most used material, with 38% of participants reporting its use. RTV silicone offers flexibility and can be useful in creating more lifelike prostheses due to its texture. HTV silicone, selected by 16.5% of respondents, is another form of silicone used in prosthetic fabrication, valued for its high-temperature resistance. The remaining 11.5% of respondents used other unspecified materials, which may point to emerging materials or regional preferences not widely adopted in the general industry.

In terms of dental material handling and understanding, an overwhelming 78.5% of respondents expressed that they felt the exercise would help them better understand and handle dental materials. This is significant as it suggests that practical experience and hands-on exercises play a key role in improving students' grasp of material properties, ultimately contributing to their skill development in clinical settings. However, 14% felt that the exercise would not provide substantial help and 7.5% were uncertain. This could be indicative of a gap in the practical application of knowledge or a lack of sufficient exposure to the materials during preclinical exercises. The survey also probed the issue of whether certain techniques and tools should be incorporated into preclinical curricula. When asked about the inclusion of special attachments in different prostheses, there was a relatively balanced response. While 36% were in favor of including such exercises, 27.5% disagreed and 36.5% were unsure. This division suggests that while special attachments can be critical for certain types of prosthetics, there may be concerns about the complexity and practicality of teaching such skills at the preclinical level. Another significant aspect explored was the inclusion of implant placement and its prosthesis fabrication in the curriculum. An overwhelming 78% of respondents felt that these topics should be incorporated into preclinical training. Implantology is becoming increasingly vital in dental practice and this data underscores the demand for dental students to gain practical experience early on in their education. Only 15.5% disagreed and 6.5% were uncertain, which further supports the idea that implant-based treatments should be a core component of the dental curriculum. The use of 3D intraoral scanners and CAD-CAM systems in preclinical curricula also received strong support, with 81% of respondents endorsing the inclusion of exercises for these technologies. Both technologies are transforming the field of dentistry by enhancing precision, reducing chair time and offering more customized treatment options. The strong support for their inclusion in preclinical curricula reflects the growing importance of digital dentistry in both clinical practice and education. Only 12.5% and 12% of respondents opposed these topics, while the rest were unsure. This suggests that there is a clear consensus on the need for dental students to become proficient in using these advanced tools to stay competitive in the evolving dental landscape. The survey among prosthodontics postgraduate students highlights that most participants are in their second year, with preclinical exercises typically taking about 9 months to complete. While a majority finds the number of exercises adequate, a significant portion views them as too extensive. In terms of learning ease, removable and complete dentures are seen as the most straightforward exercises. The Hanau Wide Vue articulator is the most commonly used and most students report having a good grasp of occlusal schemes, particularly lingualized and monoplane occlusion. The casting process in fixed prosthodontics is considered the most difficult. For maxillofacial prostheses, PMMA and RTV silicone are the most utilized materials. Most students agree that preclinical exercises enhance their understanding of dental materials. There's strong support for integrating modern technologies and clinical applications into the curriculum, such as implants, CAD-CAM, 3D scanning and dental photography. Table 1 summarizes the survey responses from prosthodontics postgraduate students regarding various aspects of their preclinical curriculum. The responses are categorized by question and presented as percentages. The questions cover topics such as the duration of preclinical exercises, adequacy of the curriculum, types of articulators used, understanding of occlusal schemes, the use of surveyors and the inclusion of modern dental techniques like 3D intraoral scanners and CAD-CAM systems in the curriculum. It also includes the respondents' opinions on the complexity of preclinical exercises and whether specific prosthodontic practices, such as implant placement and prosthesis fabrication, should be incorporated into the preclinical training. The options for each question are listed, along with the percentage of respondents selecting each option. The results of this study provide significant insights into the perceptions of postgraduate prosthodontic students in India regarding their preclinical curriculum. As dental education continues to evolve in response to technological advancements and the shifting landscape of patient care, it is essential to align educational content with contemporary clinical practices and future expectations. A substantial proportion of students (42.5%) reported that preclinical exercises take approximately 9 months to complete, suggesting a relatively lengthy preparatory phase before engaging in clinical work. However, only 37.7% of respondents felt that the number of preclinical exercises was adequate, while 34.8% considered them too extensive and 27.5% felt they were inadequate. This suggests a divide in perceptions regarding the volume and relevance of the current curriculum, likely reflecting variability in institutional practices or teaching methods. When evaluating the difficulty of specific exercises, removable partial dentures were regarded as the easiest to understand and execute (45.9%), followed closely by complete dentures (41.5%). In contrast, fixed partial dentures and maxillofacial prosthodontics were perceived as more challenging, likely due to the complexity and precision required for these procedures. The identification of tasks such as wax pattern fabrication and ceramic buildup as difficult further underscores the need for enhanced instruction and more hands-on training in these areas. These findings are in line with those of Montero *et al.* (2018) [7], who reported that students commonly struggle with intricate aspects of prosthodontic procedures, necessitating additional practical training. In terms of occlusal schemes in complete dentures, 74.9% of students reported an adequate understanding, with lingualized and monoplane occlusion being the most familiar. However, knowledge of advanced occlusal schemes, such as neutrocentric or balanced occlusion, was less widespread.

This finding highlights potential areas for curriculum expansion to ensure comprehensive knowledge and competence in various occlusal philosophies. These observations align with those of Niwatcharoenchaikul (2019) [8], who noted that while basic occlusal principles are well understood, there is limited familiarity with more specialized techniques in many educational systems, particularly in non-Western countries. The study also revealed variability in the use of articulators, with the Hanau Wide-Vue articulator (39.6%) and Mean Value articulator (25.1%) being the most commonly used. This distribution may reflect accessibility issues or institutional preferences rather than a comprehensive educational approach. Similar findings were reported by Prajapati *et al.* (2023) [9], who observed that access to advanced articulators, particularly digital systems, is often limited in dental education, restricting students' exposure to modern tools. Given the crucial role articulators play in prosthodontics, it is recommended that institutions provide broader access to a variety of systems, including digital articulators, to ensure that students are well-prepared for contemporary clinical practice. A notable majority of respondents expressed strong interest in incorporating modern topics into the preclinical curriculum, including implant prosthetics, dental photography, intraoral scanning, CAD/CAM systems and special attachments. These findings align with the global trend toward digitalization in dental education, as observed in studies by Sonkesariya *et al.* (2024) [10]. These studies emphasized the increasing recognition of the value of digital technologies in enhancing the quality of dental education and preparing students for contemporary practice. The strong desire among students to integrate newer technologies into the curriculum underscores the growing awareness of the need for digital competencies in prosthodontics. The interest in modern technologies and advanced procedures further highlights the gap between traditional training and contemporary clinical demands. Students clearly recognize the importance of not only mastering conventional prosthodontic techniques but also developing interdisciplinary skills in digital workflows, implant prosthetics and other cutting-edge areas. This finding is supported by losif *et al.* (2024) [11], who advocated for the incorporation of digital education to improve the quality and relevance of dental curricula. Overall, the findings from this study underscore the necessity for curriculum reform that is dynamic, responsive and student-centered. Educational institutions should consider integrating advanced materials, digital workflows and clinical simulation tools, while also reducing redundancy and placing greater emphasis on critical thinking and evidence-based practice. Researchers have suggested curricula must be adaptable to the changing needs of the dental profession, ensuring that students are equipped not only with foundational knowledge but also with the skills required to thrive in modern, technology-driven clinical environments.

## Conclusion:

Postgraduate prosthodontic students' perceptions of their preclinical curriculum in India, revealing concerns about the adequacy and relevance of certain exercises is of interest. There is strong support for modernizing the curriculum by incorporating digital technologies like CAD/CAM systems, 3D intraoral scanners and implant prosthodontics. The findings emphasize the need for a more integrated, student-centered approach to align education with contemporary dental practices and better prepare future prosthodontists.
